# Increasing Our Insular World View: Interoception and Psychopathology for Psychotherapists

**DOI:** 10.3389/fnins.2017.00135

**Published:** 2017-03-21

**Authors:** Patrice Duquette

**Affiliations:** Private PracticeBirmingham, MI, USA

**Keywords:** interoception, depression, anxiety, psychotherapy, interoceptive dysfunction, mindfulness, predictive coding, interoceptive inference

## Abstract

Interoception has been determined to be an elemental aspect of the neural foundations of physiological homeostasis, subjective experience, and motivated behavior. This paper reviews current neuroscience research regarding interoception and forms of interoceptive dysfunction that may result in psychopathology, focusing on depression, and anxiety, in a manner conducive to psychotherapists engaging with it to consider clinical applications. Pertinent aspects of interoceptive system processes in relation to psychopathology are addressed: Functional interoceptive ability and the forms of its expression, the difficulty of accurate measurement of such within an individual or group, interoceptive inference processes and perturbations. Predictive coding, considered in this context as interoceptive inference, a process that integrates bottom-up and top down lines of neural information emerging from the multitude of bidirectional, anatomically hierarchical connections the insular cortex makes with other cortical, and subcortical structures, will be addressed regarding its place in psychopathological formulations. Clinical vignettes will elucidate how interoceptive disturbances might present in the therapeutic relationship, supporting the evaluation and application of scientific theory, and research findings by psychotherapists. The clinical implications of this neuroscientific research have received little attention in the psychotherapeutic setting. Increasing the knowledge base of psychotherapists and furthering awareness of the functional interactions of body and brain toward the creation of healthy and psychopathological experience benefits the patient. There is immediate need for the translational expression of scientific findings into the psychological evaluation of patients, therapeutic process, and treatment. While it may seem distant and unrelated to the affective processes that occur within the psychotherapeutic exchange, neuroscience adds a unique perspective from which to observe and live such experience for the therapist and patient. With the therapeutic relationship as the backdrop, a scientific perspective will support psychotherapists' comprehension of their patients' experience and the process of change, either through direct information, or the development of different perspectives from which to observe and interact with their patients. This paper will serve not only as a guide for psychotherapists concerning this expanding knowledge base, but also a source for neuroscience researchers intent on formulating research protocols that could produce clinical benefit.

## Introduction

“*What do I feel?” Dana said, with a confounded look on her face. “I don't really know how to answer that question-every time I look inward all I can imagine is as if I am sitting inside a box looking out at the world and the box has a glass front, but inside the box my body is a wooden statue-I can see the world but the world doesn't see me. It is as if I am invisible and can't feel anything in response to the world, or if I can, I am only looking out at the world and don't have any words for any feeling, I can only sense that glass wall. Is it always going to be like that*?”

There are many different approaches to conceptualizing psychopathology within the psychotherapeutic setting. The inclusion of the patient's bodily experience is too often undervalued in evaluation of health and psychopathology by clinicians. Concurrently, as neuroscience researchers seek to include the phenomenological experience of the patient in the study of bodily-based psychopathological experience, there is not enough interdisciplinary exchange with treating clinicians to place this scientific information in a context with clinical meaning. Interoception is a neurophysiologic process that bridges the gulf between exploratory research and clinical implementation. Interoception, a vital process that sends neural information from the body to the brain, regulates life processes at the most basic levels, while also modulating emotional experience and subjective awareness at the most complex levels. It is the “process of how the brain senses and integrates signals originating from inside the body, providing a moment by moment mapping of the body's internal landscape” (Khalsa and Lapidus, 2016). As an elemental aspect of homeostatic physiological functioning, interoception substantiates the felt experience of the body, and (subjective cognitive-affective) experience, thus ultimately influencing behavior. The time has come for therapists, in our treatment of psychopathological disorders, to delve into comprehending this important concept, gaining an experiential understanding of the neurophysiologic processes that create our patients' psychological experience, and expanding our scientific and theoretical understanding of our patients and our clinical work.

Homeostasis is a process that organizes basic life processes and determines physiologic balance in the body. Craig (2015) asserts the basic purpose of homeostasis is “energy-efficient maintenance of the integrity and health of the body in support of the well-being and advancement of the individual and species.” Furthermore, Harrison et al. (2010) claim that “the central representations of organism physiological homeostasis constitute a critical aspect of the neural basis of feelings.” The interoceptive sensations arising from the body allow for a continuous monitoring and neural representation of the homeostatic state of the body through neural, hormonal, immunological, proprioceptive, and behavioral processes (Craig, 2008, 2010). Interoception, which instantiates homeostasis, is thus a vital element of healthy functioning, and disturbances in interoceptive processes on any level could create pathologic dysfunction within the individual. It is true that the complexity of the system is daunting; interoceptive dysfunction can lead to psychopathology and psychopathology can incur interoceptive dysfunction. Interoceptive dysfunction stems from different sources: (i) the interoceptive signals themselves may be dysfunctional (e.g., not getting through from the body to the brain stem), or (ii) our perception of them may be biased by disorder at neuroanatomic centers, or (iii) there may be something wrong with how we top-down interpret them. As Dunn et al. (2010) note, “It can be speculated that some symptoms or disorders lead to elevated or diminished responses in the body, whereas others are associated with better or worse perception of these bodily changes, and yet others lead to different appraisals of the significance of these changes.”

Psychotherapeutic treatment, to be effective, must disturb homeostasis on some level to allow modification and change in characteristically managed processes. As neuroscience now appreciates that the brain is in a body and the body is in the world, psychotherapists have more and new information at our disposal to address how to support an individual in the life-changing process that requires experiential instability while facilitating a new state of stability. Interoception is the progenitor of the felt sense of the body. Every meaningful therapeutic encounter must qualitatively evaluate interoceptive activity and homeostatic balance, albeit within relational evaluation rather than physiological parameters, and relay a sense of safety to the patient's body through relational consistency, compassion, interest, cues such as tone and prosody of voice, eye contact, and physical gestures. Engaging with the patient's experience at a level without words, i.e., preverbal or non-verbalized, known to the patient as the felt experience in their body, supports the evocation, and expression of experience. Comprehending the available neuroscience research is not necessary to be a psychotherapist, but it adds an immediacy, significance, and substance to the experience of treating a patient, increasing the clinician's felt sense of the relational experience, encouraging new perspectives on our patients, and deepening our respect for the difficult work of change.

Neuroscience is now trying to understand not the brain as a passive filter or assimilator of information and cues, but rather to understand brain function in terms of predictions or inferences, and how nervous system processes facilitate constructive interactions with the relational world to support change and growth. Research on the influence of top-down signals on the neuroanatomical processes that interoception initiates has opened up a whole new “world view” regarding how neural processes that predict the next moment based on prior moments generate experience on all levels for an individual, from the simplest behaviors to the most complex processes of selfhood. This is exemplified in constructs such as predictive coding and interoceptive inference, which move the discussion of interoception and homeostasis away from a bottom-up process that simply recognizes body states (Seth, 2013; Seth and Critchley, 2013; Barrett and Simmons, 2015). The circular causality between body and brain, or between physiology and mindful experience, presents a window of opportunity for both neuroscientists and clinical therapists.

Unfortunately, insufficient communication and collaboration between the research community and the clinical community is resulting in limited clinical applicability of research findings. Gallese (2014) calls for closer coordination between psychiatry and cognitive neuroscience in evaluating the cause and treatment of psychopathology, lamenting that psychiatry has been neglecting the experiential or first-person experience of the patient lately, instead “there is an over-focus on reliability (operational concepts).” Although Gallese is addressing both cognitive neuroscience and psychiatry specifically in this editorial, he defines psychopathology as stated by Jaspers (1997) to be a “disturbance of mental phenomena (Hoenig and Hamilton translation, 1997),” a definition certainly acceptable to any discipline studying and treating the many types of “disturbance” reflected psychologically in subjective and relational experience, psychophysical expression, or behavior. Gallese insists that it is imperative that researchers and clinicians work together with more purposeful and direct collaboration regarding the study, comprehension, and delineation of psychopathology, especially from the patients' experiential perspective (Gallese, 2014). This review is meant to serve as a paving stone in the development of a road connecting disciplines, leading to greater integration of psychotherapeutic process gleaned from the hours clinicians have lived with patients with neuroscience research supporting that explores basic life processes such as interoception may lead to psychopathology.

Through multiple lines of research neuroscience, is proving how interoception is a basic building block of not only physiological experience but also psychological experience (e.g., Cameron, 2002; Craig, 2004, 2010, 2015; Critchley, 2004; Singer et al., 2009; Paulus and Stein, 2010; Seth et al., 2011; Critchley and Nagai, 2012; Gu et al., 2012, 2013; Herbert and Pollatos, 2012). After a review of basic neuroscience concepts and neuroanatomy relevant to interoception, research and theory regarding interoception and psychopathology will be considered. Firstly, how differences in interoceptive perception may result in disturbed sensitivity, awareness, or report of interoceptive sensations in depression and anxiety. Secondly, proposals will be presented, modeling how interactions between interoception, higher cortical processes, and the autonomic nervous system result in subjective experience. Finally, the clinical implications of the research findings will be summarized, especially regarding interventions that are proposed to address how interoceptive disturbance can be mediated through processes that facilitate change toward healthier functioning.

This review will not address psychopathology as a disturbance in emotional processes *per se*, rather as disturbances in homeostatic processes and of embodied experience as related to interoceptive dysfunction, with feeling, somatic, cognitive, and relational disturbances a concurrent result. The intent of this paper is not to prescribe further definition of operational concepts and scripted standard of care processes. Rather, the intent is to allow an inclusion of the physical body in clinical work, if only through verbal interaction and within the therapist's clinical evaluation processes, through an understanding of how disturbance and dysfunction in the basic process of interoception, and thus the life-regulating process of homeostasis, bring patients to our door seeking help. There are generally recognized patterns of symptoms associated with depression and anxiety, although each disorder may be organized into different sub-categories. For depression such symptoms are somatic disturbances in vegetative functions, anhedonia, excessive guilt and rumination, decreased energy, and decreased psychomotor activity. Anxiety is associated with states of hyperarousal—called anxiety or panic, worry, avoidance, increased bodily tension, poor concentration, and increased apprehension.

To encourage clinicians to recognize how such research findings are relevant in everyday relational encounters, process-oriented clinical vignettes will be interjected amongst neuroscience research findings. While not defining the particulars of a patient's demographics, past history, or social or relational setting, these vignettes are intended to help clinicians through recognition and memory of similar encounters with their patients past and present, and through such associations facilitate understanding of the scientific material presented. These short passages of descriptive material are offered to trigger a picture or a sense of a patient a clinician may have treated, thus adding depth and color to the black and white nature of the research, supporting a deeper intuitive comprehension of the material, and subsequently a more meaningful translational use of such information within the clinical hour.

## Overview of interoception, homeostasis, and related neuroanatomy

In the midst of the patient's description of her father's fight with cancer, the therapist commented softly,“You really don't want your father to die, do you?” Colleen stuttered and said, “No,” and was quickly going to continue speaking. As the therapist observed that Colleen was breathing shallowly, tears had appeared in her eyes, and the furrow in her brow had just deepened considerably, the therapist asked her to pause, and inquired, “What's going on?” Colleen answered slowly, “I don't know, when you said that just now, I felt something here, (pointing to her high chest), but now it is not there.” “Gone completely?” the therapist inquired. “Well, there is some tightness and heat a little bit, but if I try to sense it any more, it's like it's gone.” “How about you don't think about it, you just feel it, like you would a sore muscle?” Colleen pauses and looks pained. “Can you say anything about it now?” She pauses again and looks slightly frustrated, “It seems like if I think about it or focus on it there, my brain shuts down, just stops.” “Ok, then don't think or try to find words, just stay with sensing it physically.” Colleen was quiet, chuckled, and said, “Well, I don't know if I really like that option, either.”

To better comprehend interoception and related scientific research, an overview of homeostasis and the autonomic nervous system (ANS) will be presented. As interoception instantiates homeostasis (Craig, 2015) and the ANS links homeostatic signals to functional output, it is necessary to have a basic understanding of such systems before looking at the bigger picture.

As you read this description of the finer levels of neural, anatomical, or perceptual processes; remember that emotional experience and awareness occur as an emergent process across systems. As Fogel (2009) succinctly states, interoceptive processes do not de facto lead to emotional awareness, “awareness emerges as a whole systems phenomenon, a consequence of these (insula, orbitofrontal cortex) and other regions of the brain and body in the interoceptive network.” The insula and related anatomic areas that are essential for interoceptive processing and homeostatic balance, and implicated in interoceptive dysfunction, will take primacy in this review.

## Homeostasis as a basic building block of experience

The term homeostasis was coined by Cannon (1939) regarding the process of how human beings maintain physiological equilibrium amidst the inherent instability of the body's internal processes and the changing circumstances of the external environment. Cannon notes this does not mean that the body reaches a stable, non-varying state; rather homeostasis is “a condition which may vary, but which is relatively constant.” He quotes the French physiologist Charles Richet, who wrote that although a “living being” must be stable so as “not to be destroyed, dissolved, or disintegrated by the colossal forces, often adverse, which surround it” (Cannon quoting Richet, 1932), it must also have an inherent instability. Maintaining stability requires that such a being must also be “excitable and capable of modifying itself according to external stimuli and adjusting its response to the stimulation.” Cannon, in his description of the functionality of homeostasis can be understood to reference the intricate dance between stability and change that we require and seek as humans for our psychological selves:

“…every complex organization must have more or less effective self-righting adjustments in order to prevent a check on its functions or a rapid disintegration of its parts when it is subjected to stress. And it may be that an examination of the self-righting methods employed in the more complex living beings may offer hints for improving and perfecting the methods which still operate inefficiently and unsatisfactorily” (Cannon, 1939).

Before research advances made in recent decades, neuroscientists would explore such “self-righting adjustments” from the perspective of the brain, and psychotherapists would consider only the experiential as relevant in the creation of necessary characteristic “self-righting methods” in individuals. Neither adequately recognized the importance of considering a functional synthesis between body and mind, or disturbances in such a synthesis resulting in psychopathology because of “methods which operate inefficiently and unsatisfactorily.” More recently, neuroscientists, moving away from a brain-centric position, have allowed constructs like feelings and experience to enter into consideration, opening a window of opportunity for both scientists and clinicians. This window places therapists in a position to exploit—and contribute to—recent shifts in neuroscience. The powerful “self-righting” processes of homeostasis with its neurally presented interoceptive underpinnings, which also directly implement the development of subjective experience, mark a critical entry point into such an engagement between disciplines.

Craig (2010, 2015) theorizes that a sense of self results from a “cortical (that is, mental) integration of salience across all conditions” at any moment in time, with homeostatic processes determining what is salient to the individual. He proposes an overarching model regarding interoceptive experience and the production of subjective awareness or “sentience.” The foundation of this model is the perception of neuronal interoceptive signals as sensations, or “feelings” (Craig, 2010). Such signals generate pain (pricking or burning pain), temperature, itch, hunger, thirst, muscle burn or ache, joint ache, sensual touch, flush, visceral urgency, nausea, among other sensations. All of these sensations are associated with an “obligatory affect (pleasantness or unpleasantness)” (Craig, 2008). At any given moment, the pleasant or unpleasant quality of such interoceptive sensations imbues this sensation with a motivation for the individual, to move toward or away from the sensation, consciously or not, while causing reactive responses in the autonomic nervous system (Craig, 2008, 2010). The responses to such motivation may be evidenced in many ways, such as beliefs, feeling states, and gross motor behaviors.

Johnston and Olson (2015) declare “feelings from the body (interoception) represent a homeostatic readout that can induce motivations to achieve homeostatic balance when needed.” As emotions are considered to be experiential states that stem from motivations regarding the positive or negative value of any internal or external stimulus, the interoceptive flow of neural signals from the body provides essential information (consciously known or not) regarding the nature and valence of such stimuli (Rolls, 1999; LeDoux, 2002; Solomon, 2008), ultimately generating responses on many levels, including behavior, always toward homeostasis. Farb et al. (2015) assert, “Maintaining desired physiological states is critical for an organism's survival, and so interoception is a powerful motivator of behavior in the pursuit of these states (Craig, 2002, 2009).” From birth onward an individual responds to motivation stemming from homeostatic imbalances which over time are expressed in characteristic responses, ultimately determining the character and personality of the individual. Therapist must deepen their comprehension of the science behind such life processes to increase their awareness and respect for the constraints the need for homeostatic balance places on the experience of our patients. Understanding the forces at play within homeostasis, such as interoception and how such forces are generating subjective experience, and ultimately behavior for our patients allows for multi-dimensional evaluation and treatment.

## Anatomical considerations regarding interoception

For a more comprehensive description of the neuroanatomic areas related to interoception see Box 1.

Box 1 **Summary description of relevant neuroanatomy.**

Interoceptive afferent fibers enter the brainstem and terminate in the Nucleus of the Solitary Tract (NTS), the Parabrachial Nucleus (PBN), and the Periaqueductal Gray (PAG). These brainstem nucleii are largely involved in homeostatic control processes. Afferent fibers continues on to the Ventromedial Nucleus of the Thalamus, and then to the posterior insular cortex (PIC), progressing through the different portions of the insula instigating and reactive to synaptic transmission from other neuroanatomical nuclei and organs. Such exchanges within neuroanatomic areas at multiple synaptic layers and levels is termed “hierarchical,” with signaling occurring bidirectionally, between areas that are next to each other anatomically, and also layers that are not immediately adjacent (Rauss and Pourtois, 2013).

Within the PIC the interoceptive pathway produces a topographical representation of the body from anterior to posterior aspects (Craig, 2015). The middle insular cortex (MIC) has connections to the amygdala and hypothalamus, and exteroceptive stimuli centers (Craig, 2011). This area integrates interoceptive signal with other inputs, e.g., ANS fibers, and “forms a combined representation of homeostatically salient features of the individual's internal and external environment” (Craig, 2011). The anterior insular cortex (AIC) has been shown to be an integrative site, representing “a common neural substrate for embodied and experiential processes” (Harrison et al., 2010). The right AIC has been explicitly implicated in the mapping of interoceptive state and response to heartbeat detection tasks (Critchley et al., 2004). The AIC is described as a coordinating site for “high level homeostatic information, perhaps about the overall state of the body, which is an important component of emotional experience and a sense of well-being” (Simmons et al., 2012).

The Anterior Cingulate Cortex (ACC) mediates the motivational and cognitive aspects of experience through connections with the AIC and autonomic effector systems and the functional co-activation of the AIC and the ACC is necessary for many aspects of subjective experience and behavior. The ACC is responsible for the motoric elaboration of subjective feelings represented in the AIC (Critchley, 2009), thus the ACC, is labeled the “motor limbic cortex” and the AIC is considered the “sensory limbic cortex” (Craig, 2015).

Interoceptive afferent fibers (afferent—from the body to the brain; efferent—from the brain to the body) originate in receptors that are situated in all tissues of the body and then travel to the brain through small diameter fibers within a layer of tissue, or lamina, in the spinal cord (Craig, 2008). The interoceptive sensations arising from the body allow for a continuous monitoring of the state of the body through mechanisms such as heart rate, blood pressure, respiration, tension in musculature, immune system reactivity, proprioceptive signals, sensual touch, visceral activity such as gastrointestinal, and genitourinary sensations (Craig, 2008, 2010). Such fibers are always active and reporting the physiological condition of the body constantly to the brain. At the level of the brainstem, the pathway becomes bidirectional, capable of receiving, and sending signals, with bidirectional (afferent and efferent) fibers of the autonomic nervous system joining the pathway. An important parasympathetic nerve, the vagus, brings sensory information from the heart, within these fibers.

In a seminal study, Zaki et al. (2012) examined the “anatomical overlap between interoception and emotion,” using a design that required participants to attend to sensations from the body while concurrently assessing their emotional experience. Objective measures were evaluated regarding the level of subjects' interoceptive accuracy using heartbeat perception tasks, and quantified activation in certain brain areas was gathered using fMRI results. The findings showed increased activity specifically in the right Anterior Insular Cortex (AIC) and Inferior Frontal Operculum (IFO) when attending to bodily experience while monitoring emotional experience. The activity in these regions also correlated with the reported intensity of emotions that the subjects reported during various trials. Zaki et al. (2012) assert the findings verify that “emotional experience is intimately tied to information about internal bodily states.” Garfinkel and Critchley (2013) point to these findings as supporting other findings that assessing one's feeling state involves interoceptive processing of changes in body state, and also correlate them to a study by Terasawa et al. (2012), that further identifies the bilateral AIC as “a neural substrate active in both the cognitive evaluation of bodily state and appraisal of self-emotion.”

The AIC is considered to integrate emotionally and motivationally salient input from closely related regions such as the ACC, orbitofrontal cortex, and striatum so as to integrate “the behavioral agent with the feeling self” (Craig, 2010). There has been some question as to whether the AIC is the actual last stop on the train regarding the development of subjective experience. Damasio (2010) and Damasio and Caravalho (2013) disagree that it is the AIC and assert “the neural substrate of feeling states is to be found first subcortically and then secondarily repeated at a cortical level” and the “subcortical level would ensure basic feeling states while the cortical level would largely relate feeling states to cognitive process such as decision making and imagination.” Thus, they theorize that while the insula has a part to play, they assert it is the brainstem and other subcortical structures functioning together that produce summary subjective experience. Barrett and Simmons (2015) also disagree with the concept of the AIC as the central anatomic structure responsible for emotional awareness and a sense of presence. In their model it is the “multiple pathways within the combined cortical interoceptive network and the ascending pathways can construct interoceptive perceptions” that summarily creates such experiences.

It is necessary to recognize the inherent value of the bidirectional transfer of Autonomic Nervous System (ANS) information occurring along the neural pathways carrying interoceptive information into and out of the brain. This arrangement quickly allows for evaluation of the environment and essentially immediate responses to stressors through ANS processes. Porges (2011) asserts such bidirectional flow of ANS signals supports “the ability to sense and regulate internal physiological state (which) is at the base of competencies in higher order behavioral, psychological, and social processes.” ANS processes are lateralized in the AIC, with the parasympathetic input represented in the left AIC, and sympathetic input in the right AIC (Craig, 2015; Johnston and Olson, 2015).

## Interoceptive ability

The day prior to Linda's session, she had received treatment for her temporomandibular joint pain from an experienced physical therapist who began treatment by helping her adjust her posture. Linda described how the physical therapist had observed her for a moment and gently moved her back, shoulders, and neck into proper alignment, and then asked how that felt. She answered, “Uncomfortable.” When the physical therapist asked “Uncomfortable, or unfamiliar?” Linda reported that she replied “unfamiliar” and “I just burst into tears.” She went on to say the physical therapist was not surprised by her tears and reassured her that happens for many people in that situation, and then said, with tears, “I had no idea I would cry so suddenly, I was just looking for relief from this tenseness and pain in my jaw, and then when I answered her question—I'm surprised at my response. How could such a little adjustment and her comment have me crying so quickly?”

The neuroscience community is working hard toward gaining consensus regarding many aspects of interoception, as elemental as a collective definition, nomenclature, and common research protocols. Khalsa and Lapidus (2016) and Farb et al. (2015) describe interoception as a process that can be broken down into different facets, e.g., attention, discrimination, detection, accuracy/sensitivity, among others. In research into interoception, such facets of interoception are evaluated as an overall level of ability to experience and report interoceptive sensation with some degree of objective accuracy. Practically this ability can then be measured and quantified in an experimental situation, using different interoceptive stimuli, e.g., heartbeat, gastric distension, or respiratory load. For example, in the experimental testing of the ability to count one's heartbeat over a certain period of time (heartbeat perception or HBP), while considered an interoceptive ability, is recognized to include such facets as attention and discrimination, which may influence its quantification when measured under different conditions. While most researchers agree that interoceptive ability can be measured as points of greater or lesser ability along different dimensions signifying objective or subjective processing, the definition, measurement, and labeling of interoceptive ability is still inconsistent in the neuroscience literature.

Garfinkel et al. (2015) highlight the extensive confusion regarding the measurement and labeling of interoceptive ability and propose a standardization of terminology. For example, they note that the terms interoceptive awareness and interoceptive sensitivity have been used synonymously and interchangeably to label an objective measure of interoceptive ability, which they purport is actually a person's ability to accurately determine the interoceptive stimulus under question. They set out to clarify distinct qualities related to the objective measure, subjective report, and subjective accuracy regarding the objective measure. To do this they measured interoceptive abilities in healthy individuals, intent on distinguishing interoceptive ability along three dimensions: Objective (interoceptive accuracy), subjective (interoceptive sensibility), and “correspondence between objective interoceptive accuracy and subjective report (interoceptive awareness).” Their tests of such processes in healthy adults using HBP tasks did distinguish between these dimensions of interoceptive ability and they assert “that interoceptive accuracy is the central construct underpinning other interoceptive measures,” i.e., interoceptive sensibility and interoceptive awareness. They further assert that the use of consistent terms that denote interoceptive ability along distinct dimensions will be helpful in discerning the clinical significance of differences in interoceptive ability to pathology. For the purposes of continuity of comparison across research studies, the terms defined by Garfinkel et al. (2015) will be used to refer to the different dimensions of interoceptive ability as tested in the research studies cited throughout this paper (see Box 2).

Box 2 **Interoceptive ability taxonomy.**

Interoceptive accuracy (IAc): Objective measurement; e.g., Heartbeat Perception (HBP) tasks, gastric distension. Garfinkel et al. (2015) note this measure is intended to “objectively quantify individual differences in behavioral performance.”

Interoceptive sensibility (IS): Subjective measurement; e.g., questionnaires; subjective scoring during a task. Garfinkel et al. (2015) assert this measurement does not necessarily relate to accuracy of perceived interoceptive stimuli rather it is the “individual's belief in their interoceptive ability and the degree to which they feel engaged by interoceptive signals.”

Interoceptive awareness (IAw): Metacognitive awareness; an equation that quantifies the amount of interoceptive accuracy relative to the subjective confidence the individual has regarding their performance on a task; as noted above evaluating whether an individual is subjectively accurate about the objective measure of interoceptive perception (Garfinkel et al., 2015) claim this “highlight(s) the relationship between subjective (perceived) and objective (actual) interoceptive ability.”

On a practical level, Schulz and Vögele (2015) claim the interoceptive perception of bodily processes requires three stages: (1) visceral **signaling** from the body to the brain, (2) the directing of **attention** toward sensation from the body, and (3) **evaluation** of such signals for their subjective meaning. Perception of interoceptive stimuli/experience is believed to develop early in life, and is considered a stable constitutional trait (Garfinkel et al., 2015) similar to temperament (Mallorquí-Bagué et al., 2016). Such perception of interoceptive experience differs greatly amongst individuals, and while fundamental to the awareness of emotional state, “a person's sensitivity to internal bodily responses may be a better determinant of emotional style, predicting one's vulnerability to emotional disorders and the capacity to regulate one's own emotional state” (Garfinkel et al., 2015).

The implications of an individual's ability to subjectively detect and objectively report interoceptive sensation related to different organ systems is under scrutiny through many different research protocols, as is how the experience of interoceptive sensations results in symptomatic disturbances in some individuals but not others. Garfinkel et al. (2015) note that “interoception interacts with cognition and emotion, making measurement of individual differences in interoceptive ability broadly relevant to neuropsychology.” The ability to sense interoceptive flows of information from the body is not consistent across individuals and measurements of such are meaningful in the consideration of the clinical implications of increased or decreased ability to perceive interoceptive sensation. Critchley and Garfinkel (2015) comment on the relative import of such studies that find correlation between HBP with other interoceptive stimuli evaluation allows “inferences about an individual's more general sensitivity to internal bodily responses and arguably, by extension, their impact on emotional processes.”

As we explore here research delineating interoceptive processes within different categories of psychopathology, memories of one patient or another will occur to an interested therapist regarding the patient's typical reactions along the dimension of interoceptive ability, considering the patient's overall characteristic propensity toward accuracy (objective), sensibility, (subjective), and awareness (whether you know how accurate you are), and how they are reflected in the patient's characteristic experience, character, and psychopathology.

## Research findings in anxiety and depression

*As Karen entered the room, she nodded hello and walked quickly to her seat, talking excitedly as she sat down. “I called the pharmacy on the way to get my father's prescriptions expecting there was one last refill on them, but nooooo, I couldn't pick it up on the way—it just messes everything up!” Although the therapist knew that Karen was the primary caretaker for her elderly father, this amount of agitation over a straightforward task seemed heightened. “**How** is that?” the therapist asked. “It is always something, I was feeling good yesterday, not so hopeless, well, now here it is again, it's all hopeless!” she exclaimed. The therapist hearing a familiar statement, and recognizing Karen was speaking from a persistent position she held about her sense of value in the world, asked, “How **is** that?” Karen replied after a moment, talking a little more slowly, “I can just see him (her father) sitting there unhappy, it feels like I have to do something to make it better for him, I'm responsible for how he feels. “I feel so depressed.” She was silently crying, looking away from the therapist. Slowly, the therapist spoke, “And, how is **that**?” Karen looked up, “You've already asked me that question,” she said warily*.

Making direct eye contact, the therapist said, “I know, can you hear how I asked the questions again in your head, do they have the same sound?” After a long pause, Karen replied, “No, they don't. How is **that**?' she repeated. “That is a place that is all too familiar, I get so scared and feel so bad, like from my body inwards I am just bad, it's like—no matter what I do I can never be a good thing in his eyes.” She paused again and said slowly, “I can feel just a bit of loosening, lightening, right here (pointing to the center of her chest), as if it is saying that how it feels just isn't true, just doesn't have to be, ‘cause no matter how it feels inside me, I am not a bad thing.”

As Bonnie witnessed another patient in group yell loudly in an expression of anger, her eyes widened, she put her hand over her mouth and looked purposefully out the window. The therapist called her name, and engaged her in conversation, asking, “What's going on, Bonnie?” She replied, “I don't know, it is just scary, just scary.” “Is it dangerous?” the therapist inquired. “I don't know, it's so loud in my head, not out here!” The therapist asked again, “Is it dangerous?” Bonnie replied, “My head says no, it says no! But my body says, just, well, it just says- I don't know and I want out! But each time I keep on finding out that it turns out ok, so I am staying now, even if I am scared.”

This review will focus on research regarding depression and anxiety. While there are differences in terminology regarding interoceptive ability, as noted above, and also different levels of Major Depressive Disorder (MDD) and subsets of anxiety within the neuroscientific literature, an attempt is made here to organize such categorization for the purposes of comparison. The chosen studies are meant to highlight interoceptive dysfunction as reflected in the expression of affective, cognitive and behavioral symptoms in these disorders.

Pollatos et al. (2007) examined the “interrelationships between experienced emotion intensity, anxiety, and interoceptive awareness” using HBP tasks. They found a significant correlation between Interoceptive Accuracy (IAc) and trait anxiety, similarly to Critchley et al. (2004). They also determined by regression analyses that IAc was a mediating factor between trait anxiety and the “experienced intensity of unpleasant pictures,” or negative feeling experience. They note such findings suggest an association between IAc and changes in body state that occur during emotional experience.

Pollatos et al. (2009) studied the association between IAc, depression, and trait anxiety, as measured by heartbeat tracking methods using counting, and depression measured by the Beck Depression Inventory (BDI). They did note that the level of depression as measured in the subjects for this study indicated only mild or moderate levels of depression but not severe depression. Within that population there was a negative correlation between HBP and depression, with higher depression scores correlating to lower IAc. They evaluated the interaction between anxiety and depression, finding that anxiety was associated with increased IAc, with the negative correlation between depression and IAc reaching significance only in subjects with high anxiety, not low anxiety.

Domschke et al. (2010) and Garfinkel and Critchley (2013) summarily note that IAc has been found to be elevated in anxiety disorders. There are varying hypotheses regarding possible cause and effect relationship of these findings. Domschke et al. (2010) propose that such an elevation might increase vulnerability to anxiety, “by increasing the perceptual base for catastrophic interpretations of cardiac symptoms,” with the increase in Iac promoting increase in anxiety through altered cognitive interpretations of sensations. Garfinkel and Critchley (2013) point out that as anxiety patients in remission even show higher than usual interoceptive accuracy (Ehlers et al., 1995), this may be because of the constitutional quality of interoception could promote vulnerability to anxiety in individuals. Interestingly, the results of Daubenmier et al. (2013) present both sides of these studies but examine the effect of life processes on such experiences, evaluating IAc in subjects who meditate. They evaluated IAc using a respiratory stimulus paradigm and heartbeat tracking, finding higher IAc to be associated with less anxiety and lower Interoceptive Awareness (IAw) by subjective report among meditators compared to non-meditators. They theorize such findings may relate to the quality of interoceptive awareness generated by such sensitivity. They theorize that a “non-evaluative” awareness of the interoceptive accuracy by the meditators may involve lower responsive anxiety and also be reflected in less subjective experience of interoception.

Furman et al. (2013), evaluating depressed patients without concurrent anxiety, found decreased Iac in subjects with MDD compared to controls. Furthermore, within the MDD group they also found that patients with lower Iac exhibited less positive affectivity and more difficulty in decision-making paradigm tasks. They state these findings indicate that for subjects with MDD, “disrupted perception(s) of bodily responses reduces both the experience of positive arousal and the ability to use interoceptive feedback to inform decision making.”

While the research findings about interoceptive ability regarding depression and anxiety are not all in agreement, there are trends noted in each disorder. IAc is generally found to be lower in depressed individuals (Pollatos et al., 2009; Khalsa and Lapidus, 2016) and higher in anxious people (Domschke et al., 2010; Mallorquí-Bagué et al., 2016). Reports of differences in insula activation are becoming more common. Initially, subjects with varying levels of pathology were grouped together, with disparate findings among groups reflecting this, especially for depression (Pollatos et al., 2009; Dunn et al., 2010). In general, evaluation of depressed subjects has found lower activation in the insula (Khalsa and Lapidus, 2016) but higher activation in anxious subjects (Alvarez et al., 2015).

Paulus and Stein (2010) consider similarities between the symptoms expressed in depression and anxiety processes and hypothesize a model of dysfunction including the insula and disturbed interoceptive that focuses on altered responses to internal body signals, or afferent interoceptive signals, due to an initial disturbance in the anticipatory state of the individual regarding what such signals mean. They assert that because of an individual's “increased bias toward negative self-view (depression) or increased attentional bias toward threat (anxiety),” the interpretation of interoceptive afferent signals is distorted relative to this bias. Paulus and Stein (2010) propose “external cues or internal thought processes generate an anticipation of aversive body states that sets up a body prediction error, i.e., the difference between the current and anticipated body state. This body prediction error acts as a motivating signal for individuals to withdraw (depression) or avoid (anxiety)” They theorize such an persistent distortion in interpreting the interoceptive flow of information accurately in relation to the present moment (and not their biased interpretation of the stimuli of the present moment) ultimately leads to the symptoms of depression (withdrawal) or anxiety (avoidance). Citing research studies that implicate the insula and related neuroanatomical areas in disturbances in self reassurance (Longe et al., 2009), worrying (Hoehn-Saric et al., 2004), anticipation of aversive events (e.g., Nitschke et al., 2006; Simmons et al., 2008) they note that “taken together, these data suggest that the insula plays an important role in processing the anticipation and subjective experience of aversive stimuli across a number of different modalities” (Paulus and Stein, 2010).

Avery et al. (2014) evaluated naturally occurring interoceptive attention to visceral experience comparing interoception in subjects with MDD and healthy subjects. They asked subjects to discern the perceived intensity of sensation from their heart, stomach, or bladder for periods of 10 s at a time while in an fMRI scanner, thus stimulating interoceptive signals from different organ systems and evaluating interoceptive perception, while simultaneously measuring the extent of activation in different neuroanatomic areas through fMRI evaluation. The fMRI results showed less activity in the dorsal mid-insular cortex (dmIC) in subjects with MDD, with a significant negative correlation of MDD symptoms to quantified BOLD signal activity in the dmIC during tasks measuring interoceptive accuracy. Thus, patients with greater symptoms of MDD had lesser activity in the dmIC. Also, specifically during HBP tasks Avery et al. (2014) found a negative correlation between insula activity, depression severity and somatic symptom severity. Thus, with greater depression and somatic symptoms they found lower insula activity. They claim such findings denote the dmIC as “a primary viscerosensory region of the insula,” which is shown in this study to be “critically affected” in MDD, possibly reflected in the findings of decreased Iac and increased somatic symptoms in MDD patients but not controls.

Kawaguchi et al. (2016) evaluated patients with social anxiety disorder (SAD) regarding any difference in insular volume from controls. The results of their study showed a significantly lower insular volume bilaterally in subjects with SAD compared to controls. Discussion of the results addressed the role of the insula in interoception and current considerations of “misinteroception” resulting in SAD patients recognizing “their somatic symptoms, such as blushing or trembling, as hazardous alarm to self, which reinforce their negative cognitions (Paulus and Stein, 2006).” Kawaguchi et al. (2016) also note the importance of the insula, along with ACC connections, in the saliency network, proposing that alterations in the insula could disturb the functioning of this network, resulting in improper grading of stimuli import and subsequent symptoms of SAD such as negative social cognitions, social withdrawal and avoidance.

A recent study by Hyett et al. (2015) concerning the symptoms of “melancholia” or “endogenous depression” is relevant regarding hypothesized functional network connectivity between the insula and other neuroanatomic areas and subsequent MDD symptoms. Using functional MRI protocols the authors evaluated differences in the functional network processes and disturbances of concentration and attention of patients with melancholia (predominant symptoms; psychomotor disturbances and anhedonia) relative to non-melancholic depression. They note such symptoms are indicative of somatic preoccupation, rumination and difficulty in shifting attention between spontaneous thoughts. Their study focused on functional connectivity between certain circuits of brain activity (called “modes”) during periods of “spontaneously generated thought” as they note “much of the illness burden is experienced through unpleasant and dysphoric affects during spontaneous thought” in melancholia. They found diminished connectivity between key networks that are important in attention and affect regulation in melancholic patients, particularly between the insula and executive mode circuit, which “includes key regions (e.g., vmPFC) subserving affective control mechanisms.”

Wiebking et al. (2015) evaluated Iac while simultaneously measuring functional MRI (fMRI) results of depressed, remission from depression, and control participants, using two distinct tasks for each group: (1) HBP counting (interoceptive) and (2) response to an external tone (exteroceptive). They found that controls and patients whose depression had remitted showed more right anterior insula activity when attending to heartbeats than when attending to external tones, while patients with depression showed no such difference in insula activity when attending to either internal or external stimuli. The authors point out that as the insula is theorized to play an important role in integrating interoceptive and exteroceptive stimuli and producing a sense of self (Craig, 2010), such a lack of differentiation in insular activity between internal and external stimuli in MDD patients may be reflected as symptoms of “altered self-awareness in depression.” Furthermore, evaluating fMRI results across groups they found reduced overall activity in the anterior insula only in the MDD group during tasks measuring IAc, which they assert might support the fMRI evaluation of insular response as a “state marker” for depression in the diagnostic evaluations of patients.

## Theoretical considerations

Robert was a medical doctor whose anxiety had always focused on his actions hurting others. For example, he imagined contracting a disease which he then would pass on to someone he loved who would die from it. After much productive work in his therapy, he could recognize his ruminations were not likely accurate, his anxiety was less, and he was better able to describe his affective experience. Upon the death of the brother with whom he was closest, his anxiety surged again, and a constant sense of fear and dread that he would miss a clinical symptom or sign, thereby harming someone caused him to ruminate frequently during his work day. He would brood over encounters with patients, finding multiple ways that his ineptness or lack of attention to detail would cause the patient harm. He was inundated constantly with feelings around the idea that he hadn't done something and that hastened his brother's death. “I just want to stop ruminating, but my anxiety just kicks it up.” In session 1 day as he spoke about his anxiety and surety that “I am going to miss something and it will be bad, and that is all I can think about the whole day” his therapist interjected “What do you feel in your body as you say that now?” Silence, and then he responded, “Something in my belly.” His therapist invited him, “Look at me and try to find more words for that feeling.” His eye contact held, tears began to well-up in his eyes, and he said slowly, “I can feel a weight dropping and dropping through me, like I am falling into a bottomless pit, it feels so real.” He continued to quietly cry and yet maintain eye contact over several minutes, his breathing ultimately deepening, his eyes lightening, and his forehead becoming less furrowed. “How is this now?” his therapist asked. “Certainly not as scary as all the time I spend alone in my head, but I can feel the line shifting constantly between feeling what is in my body and going back to that circular loop running in my head, all the while trying to see you.”

Thus far we have looked at approaches neuroscience researchers are using to address relationships between interoceptive function and psychopathological conditions. Models are being proposed to account for the interaction of bottom-up interoceptive neural signals from the body with top-down neural signals from neuroanatomic centers, producing experience on multiple levels, e.g., emotional, psychological, cognitive. While there are several choices regarding such models, we will focus in this review on the role that predictive processes are proposed to play, as they account well for homeostatic processes and the power of, “more or less effective self-righting adjustments” (Cannon, 1939) that develop over a lifetime as a person seeks to manage the inherent affective vulnerability of being human, but which can produce symptoms of depression or anxiety.

Harshaw (2015) thoroughly reviews the extant literature regarding studies of interoceptive dysfunction and depression, grouping them according to task and findings. He proposes three paths, ultimately interrelated functionally, through which interoceptive dysfunction can lead to and increase depression. These are “(a) alteration of neural substrates for interoception” whereby neuroanatomic centers responsible for interoception are disturbed through the effects of stress, and neurological disturbances, among others and, “(b) the loss of situational cues ordinarily used to disambiguate interoceptive signals, due to situational or behavioral changes, like withdrawal.” Noting that as a person's social situation from depression changes through symptomatic withdrawal and isolation, lessening available social networks, this may cause a loss of “exteroceptive scaffolding for interoception,” decreasing resources with which to distinguish bodily signals. Subsequently the depressed person may be more vulnerable to misinterpretation of both social cues and interoceptive stimuli, resulting in, “(c) shifts in *attention or awareness*, due to cognitive tendencies like analytic self-focus and rumination” (italics in original). Harshaw further addresses how other functional processes such as exteroception, the autonomic nervous system, insular function and connectivity, and social processes, immune system factors, among others, intersect at the level of interoception, and contribute to ongoing interoceptive dysfunction in depression. He claims that “focus on interoception thus provides a novel means of elucidating not only the poorly understood connection between mind, body, and psychosocial context but also the gender bias in the epidemiology of depression.”

If one considers that human beings at a basic operational level do not like uncertainty, or perturbations of homeostasis and ultimately subjective experience (Friston, 2009), neuroscience is attempting to account for how the brain and body participate to create a stable, relatively predictable perspective of inner and outer experience at any given moment through models grouped together under the rubric of “predictive coding” (Clark, 2016). The implications of such models are that the mind, or brain, while constantly making inferences or predictions about experience, is essentially trying to minimize surprise (Friston, 2009). Of course, for an infant, with very limited resources for managing the experience of physiological processes and emotion and before the ability to comprehend cause and effect, life is a constant surprise. The characteristic means employed to generate physiologic balance (homeostasis) throughout the infant's and child's varied internal and external environmental experiences may be life preserving. When such processes prevail in adulthood as patterns that are inconsistent with the reality of the moment, they often result in psychopathology that requires conscious, mindful awareness in order to change.

In depression and anxiety, a core symptom is a non-adaptive and inaccurate evaluation of internal sensations and external reality. For individuals with such disorders the evaluation of the input of their body and the external world results in characteristic experiences that create a consistent sense of distressing disorder, with fear a constant companion, and persistent beliefs that any incoming information from the self or the environment cannot be trusted. Research is slowly determining the intricacies of the neurobiologic processes that create ongoing estimations, or “predictions,” by the brain about internal states and external reality, in relevant areas such as the AIC, ACC, frontal operculum (FO), and orbitofrontal cortices. Theoretical models utilizing Bayes Theorem (see Box 3) propose how the brain determines perception and experience with accuracy or distortion regarding reality using statistical formulas, denoted as “predictive coding” (Fletcher and Frith, 2009; Friston, 2009, 2010; Friston et al., 2012). Such predictive coding models propose to account for the interaction of interoceptive neural signals from the body with top-down neural signals through such prediction-based processes to instantiate numerous aspects of subjective experience (Singer et al., 2009; Seth et al., 2011; Clark, 2013, 2016; Seth, 2013; Seth and Critchley, 2013; Pezzuolo, 2014). Current research has been focusing on proving how predictive coding processes can be integral in emotional awareness, selfhood, and other aspects of subjective experience. After a review of the research it will become apparent how interoceptive dysfunction at any level can involve disruption of the hierarchical processing proposed in such models, causing psychopathology.

Box 3 **Description of bayes theorem.**

Bayes' Theorem is a mathematical proposition that effectively summarizes the tenets of such models of perception. (Friston and Stephan, 2007) nicely illustrates Bayes' Theorem with a “prose” summation: “Given some phenomenon (A) that we want to know about, and an observation (X) that is evidence relating to A, Bayes' Theorem tells how much we should update our knowledge of A, given the new evidence X.” This update of the knowledge of A occurs repeatedly as we gain more evidence from each observation to improve the original knowledge. As a “Bayesian observer,” the brain attempts to update its knowledge regarding the phenomena of inner and outer experience analogous to Bayes' Theorem; the brain attempts to “know about” inner and outer experience, creating predictions (regarding the phenomenon A), then evaluating the result of the prediction with an incoming flow of interoceptive information (or an observation X) and making an “update” about experience (Friston and Stephan, 2007). In other words, the initial observation is considered as a prediction, and the updating of this prediction occurs by taking stock of the more recent incoming evidence from the body and “calculating” a prediction error between the prediction and afferent (incoming) information, which then may, or may not, qualitatively change the initial prediction.

The concepts of predictive coding and prediction error can be applied beyond providing an account of how the brain adjusts its internal model of how sensations are caused, creating perception. Applying such concepts to how we move can explain action and behaviors. For example, if afferent (from the body part to the spinal cord) predictions about the state of our body produce prediction errors (proprioceptive) that are eliminated by engaging classical reflex arcs (returning from the spinal cord to the body part) movement can be generated quickly. This is a simple perspective on the engagement of motor reflexes regarded as acting out afferent predictions—or when responding to predictions about how one is to move, labeled active inference (Friston, 2010).

These same active inference processes are applicable to interoception and homeostasis, where autonomic reflexes can be considered to be innately responsive to top-down (homoeostatic) predictions. Using the terms of physiology and neurobiology, physiologic balance homeostatically maintains a set point, i.e., a prediction, which is a quantifiable measure encoded in neuronal firing rates in the brain reflecting the interoceptive status of the body. Prediction error is the difference between sensory inputs from the body and descending predictions from the brain (i.e., the homoeostatic set point) that excite and inhibit certain brain cells. Thus, the change in the cell firing rate, quantified as the difference in the cell firing rate after excitation and inhibition, emerges as a neural signal and encodes prediction error. These neurally processed prediction errors subsequently ascend to higher brain centers along dedicated neural pathways to update expectations or beliefs at higher levels of representation, providing more accurate iterations of descending predictions from cortical areas (top-down) to areas receiving interoceptive input (bottom-up) from the body (which subsequently reduces prediction error). Accordingly, Khalsa and Lapidus (2016) assert that when prediction errors have been reduced to zero a body can be said to be in homeostasis. Within this description, interoception rests on sensory inputs from the body, while homoeostasis and autonomic reflexes can come to be an integral part of perception—and implicit beliefs about the state of the world, including one's own body. This is labeled interoceptive inference (Seth, 2013; Seth and Critchley, 2013).

Seth and Critchley (2013) further elaborate an “interoceptive predictive coding model” with the “anterior insular cortex (providing) a natural locus for comparator mechanisms” anatomically for the process. Seth (2013) notes within the AIC there is “evidence of substantial cross-talk between levels of viscerosensory representation, including top-down cortical and behavioral influences to brainstem and spinal centers,” with feelings/experienced emotions “hypothesized to depend on the integrated content of these predictive representations across multiple levels (Seth et al., 2011).” Seth and Critchley (2013) assert that interoceptive predictive coding, or interoceptive inference, occurs through “hierarchically cascading top-down interoceptive predictions that counterflow with bottom-up interoception prediction errors” summarily determining subjective feeling states, with behavior also resulting from such circular causality. This is because in active inference, interoceptive experience is affected by down-flowing predictions generated by perceptual content from other cortical areas that can stimulate behavior to close the gap between expected interoceptive sensation and current sensation, in an ongoing attempt to eliminate prediction error.

Seth (2013) describes two distinct functional methods by which prediction error will be resolved through interoceptive inference: “Importantly, prediction errors can be minimized either by updating generative models (**perceptual inference** and learning; changing the model to fit the world) or by performing actions to bring about sensory states in line with predictions (**active inference**; changing the world to fit the model).” Barrett and Simmons (2015) expand upon this idea of interoceptive inference, proposing various neuroanatomic areas that could be responsible for the neuronal activity that generates prediction and responds to interoceptive afferent signals, stimulating prediction errors, and the connectivity patterns underlying the hierarchical processes. The neuroanatomic complexity of such a proposal cannot be adequately summarized here, but it is notable that Barrett and Simmons (2015) agree with Seth's two listed proposals, asserting cortical connections are positioned to update generative models, and propose the thalamus, which is highly responsive to sensory input, may subsequently activate action through “signals to the motor system.” They further propose that a greater minimization of prediction error could occur through a change in the focus of attention, thus “biasing the influence of incoming sensory input” through certain network activations in the brain.

Barrett et al. (2016) address active inference explicitly in the context of depression, including concepts related to energy expenditure and management, in a theory labeled, “Embodied Predictive Interoception Coding model.” Including the concept of allostasis, the means by which the “brain efficiently maintains energy regulation of the body,” they organize the concept of prediction around how accurate initial predictions are and how effectively the brain reduces prediction error. They assert that anatomical connections within the limbic system (organized within a network termed the “salience network”) can influence the precision of the predictions, allostatically improving the efficiency of the internal model of the brain regarding interoceptive signals and the external environment. For example, salience network connections from the amygdala to the cortex can “tune” the response of the cortex to incoming predictions by signaling uncertainty, increasing precision by modulating the relative gain in neurons in various cortical areas as they compute prediction error and improving physiological regulation. In the case of depression, dysfunctional interoceptive ability through poor interoceptive accuracy, sensitivity, or awareness, and/or disordered precision through poor salience network precision weighting on cortical prediction error processing will create distorted models. Barrett et al. (2016) describe how various symptoms of depression could be the result of inefficient energy regulation and disturbed allostasis, resulting from “internal models with certain characteristics result(ing) in inefficient energy regulation (either when they are insensitive to prediction errors and/or when they are subject to poorly calibrated precision estimates).” They delineate how examples of early life stressors such as neglect, abuse or limited positive interactions, would require larger expenditures of energy and establish a model of the world that is constantly predicting and reacting to internal (e.g., fatigue, poor nutrition, or physical illness) or external stressors (e.g., social interactions or isolation) with decreased metabolic efficiency and poorly regulated energy. Symptoms such as negative affect, withdrawal, fatigue and poor sleep may result from such “chronic energy inefficiency and altered interoceptive signaling” processes (Barrett et al., 2016).

Pezzuolo (2014) presents a predictive coding theory which incorporates interoceptive, perceptual, and cognitive inference processes, labelelled “embodied predictive coding,” in an engaging paper that proposes how physiologic sensation and subsequent affective experience, might overcome the rational mind when evaluating reality, sometimes fearfully creating a “bogeyman” out of whole cloth when there is actually no danger. Noting “in most cases interoceptive information is quite certain, so it has a greater influence on the inference,” he notes the relative weight given to interoceptive signals at any given moment can distort the predictive coding processing of stimuli from all sources. He terms this process “embodied predictive coding.” He describes how such dynamic processes can result in experience and behavior that reflects the reality of the moment, while at other times can create significant reality distortion, reflected in pathological disturbances.

Gu and FitzGerald (2014) describe the value of interoceptive inference toward minimizing surprise and maintaining homeostatic balance in the context of decision making and motivated behavior. They note that “organisms” (we address the human organism here), seek “out the states they expect to occupy, where these “familiar” states are innately valuable (Friston, 2010),” as such familiarity inherently lessens surprise. Thus, in support of homeostasis during “non-familiar” states, autonomic reactions are instigated by active inference processes, and the organism can also perform actions on the world so as to bring tits internal milieu back to homeostatic balance, decreasing prediction error in interoceptive inference processes. They assert that such actions are initiated by interoceptive inference processes, thereby informing the organism regarding “value-based choices about the internal state of the (their) body.” Psychotherapists will recognize behavior resulting from such “choice” as either intent on self-care, or to use psychotherapeutic jargon, “acting out.”

Finally, in an imaginative article that presents a discussion between a philosopher, a theorist and a physicist (Friston et al., 2012) in which each writer applies their discipline to address a neuroscientifically-based theoretical proposal that “all biological systems are driven to minimize “free energy”” (Friston and Stephan, 2007; Friston, 2010). Free energy can be conceived of as surprise, or in psychotherapeutic terms, uncertainty or fear. While the discussion is imbued with mathematical, scientific and philosophic terms, the underlying “music” of the discussion at hand echoes the work of psychotherapy; how do we help our patients as they struggle to change their model of the world to be less driven by characteristic responses to fear. Several statements by the physicist (Friston) supports exploration of this seemingly complex world of neuroscience by interested therapists. He writes:

“Avoiding surprises means that one has to model and anticipate a changing and itinerant world. This implies that the models used to quantify surprise must themselves embody itinerant wandering through sensory states (because they have been selected by exposure to an inconstant world): Under the free-energy principle, the agent will become an optimal (if approximate) model of its environment” (Friston et al., 2012).

The infant models their world as best they can while swimming in a sea of sensation, and through persistent efforts at “pushing away from fear and dread” (Bar-Levav, 1988), such “agents” (Friston et al., 2012) will develop models that are expressed in characteristic perspectives and behaviors that reflect the “model of its environment” they have created. Friston et al. (2012) echoes statements by neuroscientists earlier in this paper (Seth, 2013; Barrett et al., 2016) regarding how human beings continue on while remaining inherently vulnerable to the vagaries of life, “surprise can be reduced by changing sensory input (action), predictions of that input (perception), or the model *per s*e.” The “physicist” (Friston) also asserts that “Evolutionary or neurodevelopmental optimization of a model is distinct from perception and entails changing the form and architecture of an agent.” Such language addresses the ultimate goal of psychotherapy regarding therapeutic change reflected in changes in brain function, evidenced by our patients living more emotionally open and realistically in the face of the uncertainty of life. We must not only help our patients “feel” differently, but must support actual changes in brain function through relational interaction that will have them live differently, with more realistic models of the world and reactions fitting to such models.

## Conclusion

“*Oh,” Celeste said with a gasp of tears, as she placed her hand in the middle of her chest, “Oh, this just feels better.” She was in her group, and had just been speaking about various issues in her marriage that troubled her. Her therapist inquired, “What is going on?” Celeste answered, “It just feels better to put my hand here, over the part that is cold and can't be felt. It is so different, it used to be my whole chest was cold and numb; now it is just this circle right in the middle of my chest that is cold. It just feels like I am holding it, that piece of cold, and as I put my hand here it feels better. It's like my body is thawing, especially my heart.”*

All of life that one encounters is perceived and responded to within one's body. It is proposed that subjective experience results from hierarchical processing of stimuli from the body and the environment, through complex neural systems inferring the cause of such stimuli and creating cohesive “explanations” of such stimuli, with ensuing physiologic homeostatic regulation. Patients often enter psychotherapy when over time such methods fail more than they succeed to regulate their physiology. Dysfunction in the processing of bodily stimuli, considered in this review as interoceptive dysfunction, has been evaluated along various research based dimensions regarding how such dysfunction may present as symptomatic depression and anxiety. Now, consideration will be now made of various means psychotherapists have available through relational interactions to evaluate and include such research as a backdrop in clinical interventions. Fogel (2009) uses the term “willingness to be a process” to describe a vital characteristic developed in psychotherapy. Neuroscience also recognizes the elemental aspect of process, and is reflected in research that supports the idea that the work of psychotherapy is the experiential and relational evaluation of perceptions and implicit beliefs. Such a view of psychotherapeutic process could be readily stated in Bayesian terms as the evidence-based updating of prior beliefs, and persistent efforts to lessen the distorting influences of feelings on perception, cognitions, and behavior.

The form of the basic underlying physiological, emotional, and/or cognitive processes expressed by our patients' bodies originates within early attachment relationships and echoes within their experience throughout their lives. It is these echoes which psychotherapists listen for, visualize, and imagine, sitting with their patients, that is, such echoes are the “stuff” of experiential connection, and can potentially become “audible” to us and them, through the relational work of therapy. Sitting across from our patients we can train an evaluative eye on the level of function in these processes from their outward manifestations, noting, for example a patient's breathing, facial musculature, posture, prosody and pitch of voice, eye contact, limb movements, or the look in their eye as they make eye contact or not. Van Der Kolk (2014) highlights the import of the therapist gaining awareness of bodily experience for themselves and for their patients and its expression in experience. “We can get past the slipperiness of words by engaging the self-observing, body-based system, which speaks through sensations, tone of voice, and body tensions.”

The literature cited regarding interoceptive dysfunction points to the importance of interoceptive experience that is bounded by expectations (or predictions) that the world will be a beneficent place or at least not maleficent, otherwise there is a much greater likelihood of symptomatic experience. Regarding anxiety, studies cited propose that high interoceptive accuracy stimulates an increased likelihood of “catastrophic interpretations” of physical symptoms (Domschke et al., 2010), with panic an all too likely consequence. Considering the same dimension of interoceptive ability regarding depression, low interoceptive accuracy is reflected in symptoms such as disruptions in decision making and low positive affect (Furman et al., 2013).

On the face of it, the likelihood of increased perception of interoceptive sensations leading to anxiety states, and lower perception of interoceptive sensation leading to despairing states seems counterintuitive, as the commonplace description of these disorders does not immediately appear to reflect this. It is commonly considered that the symptoms of anxiety occur when a person is caught unawares by strong bodily sensation with sudden anxiety or “panic attacks,” while the person who is depressed strongly experiences body-based vegetative symptoms of depression and is overwhelmed by the strength of such feelings. Yet such research findings may reflect, in an operational sense, the inherent need human beings have to create reasons for the causes of any experience to limit uncertainty, whether the reason is realistic, or not. As Pezzuolo (2014) notes, “*interoceptive information is part and parcel of the representation of entities*” (italics in original), a statement which reflects the expression of the interweaving of interoceptive experience and uncertainty in top-down prediction of cause, and all efforts to decrease the unknown by the mind. Clark (2016) also comments on this statement by Pezzuolo (2014) in a footnote, remarking that “internal states that become active in the presence of specific external states of affairs are always richly contextually inflected,” since any context for an individual is replete with past and present experiences that can be known or unknown in any present moment, because, as Clark asserts, “this inflection now seamlessly combines “objective” and “subjective” (e.g., emotional and body related) elements.”

The clinical implications of this neuroscientific research have received little attention in the psychotherapeutic setting. Increasing the knowledge base of psychotherapists and furthering awareness of the functional interactions of body and brain toward the creation of healthy and psychopathological experience benefits the patient. There is immediate need for the translational expression of scientific findings into the psychological evaluation of patients, therapeutic process, and treatment. While it may seem distant and unrelated to the affective processes that occur within the psychotherapeutic exchange, neuroscience adds a unique perspective from which to observe and live such experience for the therapist and patient. With the therapeutic relationship as the backdrop, a scientific perspective will support psychotherapists' comprehension of their patients' experience and the process of change, either through direct information, or the development of different perspectives from which to observe and interact with their patients.

The clinical vignettes presented highlight the interplay between the effects of interoceptive disturbances and the tightly held predictions reinforced by belief, past history, and current sensation that seems boundless. Such verbalizations and behaviors are exhibited by people when they feel safe enough to express their experience and take a chance with another to find out if their certainty about any aspect of the experience is accurate. The resources that therapists have available to help a patient discern safety within the relationship are sometimes deceptively simple but activate the body to assess safety, not insist on danger. One such resource is suggesting the patient breathe regularly, possibly with a longer exhale than inhale. Such a process activates the parasympathetic branch of the ANS (Porges, 2011), and controlled, slowed, breathing has been shown to decrease negative affect (Zautra et al., 2010). A suggestion that the patient place their feet on the ground would facilitate proprioceptive receptors which would send stimuli to the mid-insula, which integrates salient internal and external environmental features (Craig, 2011) engendering experience of a safe physical place and supporting a sense of real stability, that could lessen the power of other bottom-up sensation to create “noise” in a system seeking clarity. Finally, eye contact, a requisite activity in any therapeutic encounter, which Baltazar et al. (2014) found increased the accuracy of subjects' rating of their emotional reaction “with respect to their interoceptive signals” and is also proposed by this group to promote increased “self-focused attention,” can substantially encourage openness in the patient. Within the context of safety, the patient can express strong feeling, and as Fogel (2009) affirms, “feeling one's pain or fear in the subjective emotional present activates the homeostatic recovery system of the body so that it has the opportunity to take care of itself.”

Mindfulness, or the practice of meditation or other contemplative practices, is under evaluation as a means to affect functional change in interoceptive experience and inference processes in patients exhibiting depression or anxiety. Vago (2013) addresses the present moment attention mindfulness requires and claims it can “enhance capacity for the practitioner to act congruently with one's right intentions, direct perceptions, and intention-focused goals (Kabat-Zinn, 2005; Brown et al., 2007).” Mindfulness Based Stress Reduction (MBSR) is a process often taught in manual-based courses, but meditation can take many forms. While the process of how meditation works to decrease anxiety or improve mood is still under active discussion, Holzel et al. (2011), review research concerning the insula, and find increased activation in individuals after training in MBSR, and when they were “focused on their momentary experience (Farb et al., 2007).” While noting that other studies found increases in activity in the insula and thalamus under different conditions, they report that “The enhanced sensory processing has been suggested to represent increased bottom up processing of the stimulus, that is, awareness of the actual sensation of the stimulus.”

Using predictive coding as a backdrop, Farb et al. (2015) discuss the potential positive effects of contemplative practices on interoceptive inference processes, explicitly with respect to active inference and perceptual inference (NB: Perceptual inference: Changing the model to fit the world; active inference: Changing the world to fit the model; Seth, 2013). They note that the immediate nature of active inference allows for “human beings to flexibly and dynamically adapt to the world in which they are intrinsically embodied.” Alternatively, perceptual inference presumably has a different extended time course that allows for increased “ability to notice specific details of internal sensory experience such as the subtle changes in sensation,” and subsequent revolutions of these details through the predictive coding hierarchy could lead to a more accurate interpretation of the individual's sensory experience of the moment. They state that defusing the more immediate regulation of interoceptive information via active inferential processes (e.g., with contemplative practices), and allowing “iterative cycling between perceptual and active inferences,” promotes more adaptive behavior results for the individual (Farb et al., 2015). Such would be the goal of psychotherapy practice in any theoretical model, with the lessening of characteristic reactive responses and moment-to-moment awareness increasing thoughtful and flexible responses constituting improvement.

Neuroscience researchers are evaluating how mindful attention to experience in varied contexts may create benefit reflected in increased ability to self-regulate and purposeful efforts at decreasing maladaptive behaviors. Gard et al. (2014) examined the effect of various aspects of yoga practice on the promotion of psychological health. They address how yoga practice encourages purposeful focus on the experience of the body, with an “emphasis toward processing bottom-up information.” They assert that this could result in “greater precision of afferent signals as the result of increased sensory attention,” which they purport would increase perceptual inference processing of prediction errors in support of learning, and “thereby lead to extinction of maladaptive behaviors.” Huffziger et al. (2013) explored the effect of mindful attention (described as above by Vago, 2013) compared to ruminative attention in healthy young adults. While the effects of the study design considered here may appear be self-evident to any therapist, they do lend support to the expectation that seemingly minor interventions will generate more significant effects over time for our patients. Subjects used hand-held electronic devices carried throughout their day to record their subjective experience after 3 min periods of mindfulness or ruminative self-focus on alternating days. A notable finding was that the 3 min mindful attention period was followed by no change in mood valence, but “immediately enhanced momentary calmness.” This finding reflects a significant gain for the young adult subjects of the experiment as they are developing habits that could more readily persist through life. Also, since mindfulness of experience is a goal with any patient, through an open-hearted, self-observant, and self-accepting internal posture, such a finding is meaningful for a psychotherapist regarding the potential effects of a persistent tending to our patients efforts at self-care. If 3 min periods in a busy day can support calmness in treatment naïve individuals, the support of efforts to slowly but surely generate habits of such purposeful attention can benefit our patients.

Habitual patterns, predictions or inferences are necessary whether an individual is reaching for the food on a plate in front of them, walking down a hallway, attending a party, asking for a raise at work, or relating within an emotionally intimate experience. Homeostatic state, interoceptive signaling, motivational salience, prior experience, and current exteroceptive data from the environment are elements that create a template for these patterns. Muted awareness of bodily processes below the head necessarily breeds muted awareness of life itself. Thus, it is critical for a psychotherapist to consistently support their patient's evaluation of the psychotherapeutic relationship for safety, then resolutely push on into experiences that cause disruption in their homeostatic balancing processes through active questioning of their interoceptive experience and predictive modeling, both of which may be mired in the past and not allow for the fullest experience of any present moment.

Psychotherapeutic theories and processes have much to gain from neuroscience research, deepening the process of change we witness in our patients as we live with them through many difficult yet brave encounters with themselves. And neuroscience research can be enriched by examining how the psychotherapeutic relationship reshapes physiological and psychological processes to more accurately and fully correspond to the present moment. Not only by measurements of grouped individuals but the undertaking of dialoge with practitioners involved with individuals in the intimate, life changing process of psychotherapy. Neuroscientific theory and research could gain focus through engagement with clinicians who are exploring human subjective experience through personal work and the relationship with their patients, intent on creating physiologic change and the means to materially discern the “stuff” of predictions, which, concurrent with other important reparative processes, changing interoceptive process gone awry on many levels, stimulating health in the moment to moment experience of life.

## Author contributions

The author confirms being the sole contributor of this work and approved it for publication.

### Conflict of interest statement

The author declares that the research was conducted in the absence of any commercial or financial relationships that could be construed as a potential conflict of interest.
